# Return to the unknown normal: transition of team-based learning from a COVID-19 enforced online version to an on-site version

**DOI:** 10.3389/fmed.2026.1712200

**Published:** 2026-02-11

**Authors:** Teun J. de Vries, Keith Groot, Denise E. van Diermen, Geerling E. J. Langenbach, E. Etienne Verheijck, Gerard W. G. Spaai

**Affiliations:** 1 Departments of Periodontology, Academic Centre for Dentistry Amsterdam, University of Amsterdam and Vrije Universiteit, Amsterdam, Netherlands; 2 Education, Academic Centre for Dentistry Amsterdam, University of Amsterdam and Vrije Universiteit, Amsterdam, Netherlands; 3 Oral Medicine, Academic Centre for Dentistry Amsterdam, University of Amsterdam and Vrije Universiteit, Amsterdam, Netherlands; 4 Orofacial Anatomy, Department of Oral Pain and Disfunction, Academic Centre for Dentistry Amsterdam, University of Amsterdam and Vrije Universiteit, Amsterdam, Netherlands; 5 Teaching and Learning Centre, Faculty of Medicine, University of Amsterdam, Amsterdam University Medical Centre, Amsterdam, Netherlands

**Keywords:** active learning, face-to-face learning, student engagement, TBL online, TBL on-site, team-based learning

## Abstract

**Background:**

During the Covid-19 lock-down, educational institutions had to implement novel educational formats in an online form without having any prior experience with the on-site counterparts. These online formats then had to transition to an on-site format once the lockdown restrictions were lifted. Here, we describe a dentistry faculty’s transition of online team-based learning (TBL), a form of active learning initiated during Covid-19, to an on-site format.

**Methods:**

To analyze students’ and teachers’ experiences and possible preferences for one of the formats, we adopted a mixed methods research approach, using both questionnaires and interviews. Students were from the second year of the Bachelor program. They started university when TBL and all classes were delivered online. Following this online year, they had spent one academic year attending fully on-site TBL after the Covid-19 restrictions were lifted. Teachers’ initial training and experience with TBL was also online followed by on-site. Both groups filled in a 1–5 Likert scale questionnaire on various aspects of TBL, with paired questions comparing the online vs. on-site format. Both questionnaires addressed the three phases of TBL (preparatory self-study phase, readiness assurance phase (RAT), and application phase) as well as the teacher’s role. Additionally, the student questionnaire included questions related to their engagement. Complementary structured interviews with students and teachers were chosen to provide deeper insights into these topics. A paired *t*-test was used to analyze the outcomes of the 1–5 Likert scale questions. A Wilcoxon signed rank test was used as a ranked post-test. All recorded interviews were transcribed verbatim and analyzed using an inductive thematic analysis approach.

**Results:**

Out of 134 s year bachelor students 54 filled in the questionnaire (response rate = 40%). Out of 19 TBL teachers 11 teachers filled in the questionnaire (response rate = 57.8%). Two group interviews with teachers (*N* = 5) and three individual interviews with students were carried out. The questionnaire data showed that the teachers were more vocal about their preference for the on-site format than students. When analyzing the outcomes per theme, both students and teachers favored the on-site version of the readiness assurance phase. Furthermore, teachers clearly preferred the on-site application phase (*p* < 0.05). Students experienced more engagement in the on-site format. When asked about their overall preference, 66% of the students and 72% of the teachers preferred on-site TBL, no teacher preferred online TBL (*p* < 0.0001). The interviews revealed that students appreciated the possibility to follow classes without having to travel to university in the online format. While discussions were easier to perform on-site, both teachers and students valued the serenity of group work in the online breakout rooms compared to the noisier TBL lecture hall and saw this as a positive take-away of the online version. Furthermore, teachers valued the ease of sharing information during the TBL mini-lecture. Finally, teachers noted that on-site teaching allowed them more control during the application phase.

**Conclusion:**

After their experiences with the online version of TBL, both students and teachers adapted organically to the on-site version. Overall, both students and teachers appreciated a familiar environment for interactions, which is essential for active learning.

## 1 Introduction

The Covid-19 pandemic forced a rapid unprecedented shift from on-site to online learning (1, 2) requiring all educational innovations planned for the academic years 2020–2021 and 2021–2022 to be implemented in an online format. When on-site education resumed, educational institutions with prior on-site experience could easily return to campus and revert to existing routines, while those that had implemented innovations in an online format only had to develop new approaches for on-site formats and had to establish new norms, the new but unknown normal.

The Academic Centre for Dentistry Amsterdam (ACTA) experienced such a transition from online to on-site without such reference point when implementing Team-Based Learning (TBL). ACTA adopted TBL as a structural component of all courses of the Bachelor program (3) from September 2020 onward. TBL is an active learning and small group instructional strategy that provides students with opportunities to apply conceptual knowledge (4). TBL can stimulate deep learning and foster the development of health-related skills such as clinical reasoning, problem solving and collaboration (5, 6). Although TBL was initially only implemented for the first year, the implementation gradually expanded to the first 2 years, and finally to all 3 years of the Bachelor program, as an example, see (7). After 2 years, it was replaced by an on-site format.

Three studies have explored students’ experiences of TBL in an online format during the pandemic (3, 8, 9). De Vries et al. (3) found that students appreciated the teamwork in online TBL (3). Furthermore, it was found that online TBL promoted participation and active engagement through student-led discussions (3, 9). Finally, Govindarajan and Rajaraguparthy (8) found that TBL facilitated interactions among students during the pandemic.

Other studies have investigated the transition from on-site to online TBL during the pandemic. In a study by Yu et al. (10), students valued the online and the on-site TBL format equally. However, another study among pharmacy students (11) showed that students were more at ease with the new online situation than teachers, who felt less confident in delivering TBL online. Furthermore, both students and teachers in this study mentioned technical problems, the changes in delivery and the lack of student engagement, as well as the difficulty in focusing during class as the main hurdles of the transition to online TBL. Finally, Anas et al. (12) found no difference in student performance between online and in-person TBL sessions during the pandemic.

Few studies have analyzed the transition from an online to an on-site format. For example, Stoian et al. (13) conducted a study primarily from the students’ perspective, finding that they valued the availability of online lectures and considered communication to be comparable between online and on-site settings. Along with the mass return to education in academia, many institutions, among them ACTA, decided that all course activities should be on-site again, bringing a new sense of belonging to students that had been devoid of social intercourse during the Covid-19 pandemic. It was therefore necessary to also develop TBL in an on-site format.

Although many educational institutions must have introduced some online educational innovations during the Covid-19 pandemic, the transition ‘back to normal’ without having any previous experience with an on-site version has hardly been studied. This investigation aims to address this transition by answering three main questions:

1. How did students and teachers experience the transition from TBL online to TBL on-site?
2. To what extent have students and teachers experienced challenges in the transition of TBL online to TBL on-site?
3. Which effective online TBL practices identified by teachers and students could inspire the on-site version of TBL?

## 2 Materials and methods

### 2.1 Research design

A mixed-methods design (14) was employed to answer the research questions, using both questionnaires and semi-structured interviews with students and teachers. The study was carried out in two steps: quantitative data was collected through a survey with students and teachers, which was then complemented with qualitative insights from interviews about the outcomes of the surveys.

### 2.2 Participants

#### 2.2.1 Students

All 134 s year dental students, academic year 2022–2023 of the undergraduate program of the Academic Centre for Dentistry Amsterdam (ACTA) were invited to participate in the study via e-mail. Only these students had experience with both the online and the on-site version of TBL. Additionally, we used purposeful sampling and snowballing to recruit participants for the interviews. All students invited for the interview had 2 years of experience with TBL. Participation in the study was on a voluntary basis.

#### 2.2.2 Teachers

Teachers (*N* = 19) with experience in both the online and the on-site version of TBL were invited to participate in the study. All invited teachers had at least 2 years of TBL experience. Participation was on a voluntary basis.

### 2.3 Instruments/procedure

#### 2.3.1 Questionnaires

The teacher and student questionnaires were designed so that each question about the online TBL version was paired with a question about the on-site version. The questionnaires were inspired and modified from a previous study (15). The questionnaire covered the three phases of TBL: the preparation phase (i.e., self-study), the readiness assurance phase (i.e., individual Readiness Assurance Test (iRAT), team Readiness Assurance Test (tRAT), and a mini lecture focusing on concepts that are misunderstood), and the application phase (i.e., doing the applications in small groups). The questionnaires further focused on different aspects of the teachers’ role and on student engagement.

Questionnaires were similar for both students and teachers, except for the questions regarding student engagement, which were only asked in the student questionnaire.

The student questionnaire included 37 items while the teacher questionnaire included 30 items related to the online version and on-site versions of TBL. The questions were paired and structured so that each group of questions was answered first for the online variant, followed by the same questions for the on-site variant (Tables 1, 2). Each item consisted of a statement that was answered using a 5-point Likert scale, where 1 indicated complete disagreement and 5 meant complete agreement.

**Table 1:** Questions and results of the student questionnaire regarding TBL preference online vs. on-site.

Question	Online TBL	On-site TBL	*p*-value
	Mean (*n*)	Mean (*n*)	
Preparatory self-study TBL			
1. The self-study assignments helped me better understand the study material	3.8 (41)	3.7 (41)	0.84
2. The self-study assignments prepared well for the digital iRAT and the online tRAT/iRAT and tRAT in the TBL room	3.9 (41)	3.6 (41)	0.71
3. The self-study assignments prepared well for the online/onsite TBL Application sessions	3.7 (40)	3.7 (40)	0.87
4. I grade the preparatory self-study required for online/on-site TBL as…	3.6 (42)	3.5 (42)	0.64
iRAT on-site, tRAT online/iRAT and tRAT on-site			
5. The technology used to conduct the iRAT online/on-site was user-friendly	3.7 (37)	4.0 (37)	0.06
6. The breakout/TBL room was suitable for conducting the iRAT online/on-site	3.9 (35)	4.1 (35)	0.16
7. The technology (Zoom for online/on-site technology and TBL room for on-site) was suitable for conducting the tRAT	3.4 (36)	4.0 (36)	0.12
8. The tRAT encouraged me to prepare well	3.6 (36)	3.7 (36)	0.56
9. In the breakout/TBL room, we were able to discuss well together as a team about the tRAT	3.5 (35)	4.1 (35)	0.11
10. Taking the iRAT and tRAT caused me stress	3.3 (36)	3.0 (36)	0.16
11. The iRAT and tRAT questions aligned well with the self-study assignments	3.8 (36)	3.9 (36)	0.62
12. The iRAT and tRAT questions aligned well with the application tasks	3.4 (36)	3.8 (36)	0.09
13. The mini-lecture helped me understand the study material better	3.3 (36)	3.7 (36)	0.07
14. The mini-lecture was a useful part of TBL	3.3 (36)	3.7 (36)	0.20
15. I grade last year’/this year’s RAT phase (bachelor’s 1)/(bachelor’s 2) as.	3.1 (37)	3.6 (37)	0.19
Application phase			
16. The application exercises aligned well with the learning objectives of the TBL meetings	3.4 (29)	3.6 (29)	0.46
17. The application exercises helped me understand the course material better	3.6 (29)	3.6 (29)	1.00
18. The application exercises encouraged teamwork	3.6 (29)	3.5 (29)	0.86
19. The plenary discussion of the application exercises (defending answers by teams) was educational	3.5 (29)	3.5 (29)	0.55
20. The online/on-site TBL tired me out, making me less able to actively contribute	3.1 (28)	3.5 (28)	0.42
21. The Zoom environment/TBL room in which the application tasks were carried out was (well) suited for this purpose	2.8 (29)	3.6 (29)	0.14
22. The Zoom environment/TBL room contributed well to working in teams	3.1 (29)	3.8 (29)	0.11
23. I had no privacy issues during online/on-site TBL	4.0 (29)	4.1 (29)	0.86
24. Technology/the use of microphones supported the process during the application exercises	3.8 (29)	3.8 (29)	1.00
25. Teachers and students were easy to understand during online/on-site plenary moments	3.7 (29)	3.9 (29)	0.41
26. I grade the application phase of TBL online/on-site as.	3.4 (28)	3.3 (28)	0.90
TBL teachers			
27. The guidance from teachers during the online/on-site TBL meetings was good	3.6 (29)	4.1 (29)	0.20
28. The TBL teacher gave good feedback during the online/on-site TBL meetings	3.4 (29)	3.9 (29)	0.14
29. The TBL teacher encouraged active student participation during the online/on-site TBL meetings	3.4 (29)	3.8 (29)	0.16
30. TBL teachers encouraged discussion during the online/on-site TBL meetings.	3.7 (29)	3.9 (29)	0.45
31. In general, I grade the role of the TBL teachers as.	3.4 (29)	3.8 (29)	0.29
Student engagement TBL			
32. Most students actively participated during online/on-site TBL	2.9 (30)	3.8 (30)	0.02*
33. I had fun during online/on-site TBL	3.0 (30)	3.5 (30)	0.18
34. I contributed meaningfully to the discussions during the online/on-site TBL meetings	3.7 (30)	3.9 (30)	0.38
35. I actively participated during online/on-site TBL most of the time	3.5 (30)	3.5 (30)	0.78
36. I enjoyed the flow of the online/on-site TBL (RAT phase and application phase)	3.2 (30)	3.6 (30)	0.23
37. The involvement of me and my fellow students during TBL online/on-site I would grade as.	3.1 (30)	3.8 (30)	0.06

Averages of 1–5 Likert scales (1 = completely disagree to 5 = completely agree) are shown. *p < 0.05.

**Table 2:** Questions and results of the teacher questionnaire regarding TBL preference online vs. on-site.

Question	Online TBL	Onsite TBL	*p*-value
	Mean (*n*)	Mean (*n*)	
Preparatory self-study TBL			
1. Self-study assignments are designed to help students better understand the study material (online/on-site)	3.5 (11)	3.5 (11)	1.00
2. The self-study assignments are designed to prepare students well for the iRAT and the online/on-site tRAT	3.7 (11)	4.0 (11)	0.15
3. The self-study assignments are designed to align well with the online/on-site TBL application exercises	3.9 (11)	4.0 (11)	1.00
4. I graded last year’s preparatory self-study as.	3.4 (10)	3.6 (10)	0.42
iRAT on-site, tRAT online/iRAT and tRAT on-site			
5. The technology used to conduct the iRAT online/on-site was user-friendly	2.8 (9)	3.9 (9)	0.01*
6. The TBL room was suitable for conducting the iRAT online/on-site	3.0 (9)	3.3 (9)	0.57
7. The transition from iRAT to tRAT went smoothly	2.7 (9)	3.9 (9)	0.02*
8. The technology used to conduct the tRAT (Zoom/TBL room technology) was user-friendly	2.8 (9)	3.9 (9)	0.03*
9. The online/on-site iRAT and tRAT matched well with the self-study assignments	3.7 (9)	4.2 (9)	0.04*
10. The online/on-site iRAT and the tRAT matched well with the online TBL application tasks	3.7 (9)	4.2 (9)	0.04*
11. The technology used to conduct the mini-lecture was effective	3.1 (9)	3.0 (9)	0.94
12. The online/on-site mini-lecture formed a useful part of TBL	3.0 (9)	3.3 (9)	0.52
13. I grade last year’s/this year’s RAT phase (bachelor’s 1)/(bachelor’s 2) as.	2.7 (7)	3.9 (7)	0.05
Application phase			
14. The online/on-site application exercises aligned well with the learning objectives of the TBL meetings	3.8 (9)	3.9 (9)	0.77
15. In my impression, the online/on-site TBL application exercises encouraged teamwork	4.0 (9)	3.8 (9)	0.75
16. The plenary discussions on the online/on-site TBL application tasks (=defending answers by teams) went well	3.1 (9)	4.0 (9)	0.05
17. As TBL teacher, I had a valuable contribution to the online/on-site discussion phase of TBL by appearing in breakout rooms/in the TBL room	3.3 (9)	4.2 (9)	0.05
18. Students’ answer choices, given in the chat/letter with different color, were clear to me	3.0 (9)	4.6 (9)	0.01*
19. My role as a TBL teacher during the discussion phase of the application exercises (via breakout rooms/visiting the teams) went well for me	4.0 (9)	4.2 (9)	0.59
20. My role as a TBL teacher during the plenary moments went well for me	3.7 (9)	4.3 (9)	0.09
21. The Zoom environment/TBL room was a suitable environment for performing TBL application exercises	3.9 (9)	4.0 (9)	0.06
22. The Zoom environment/TBL room contributed well to working in teams	3.4 (9)	4.0 (9)	0.59
23. Teachers and students were easy to understand during online/on-site plenary moments	3.7 (9)	3.7 (9)	1.00
24. I grade the application phase of TBL online/on-site as.	3.2 (9)	4.2 (9)	0.07
TBL teacher			
25. The nice thing about online/on-site TBL was that you can easily visit the teams	3.6 (11)	4.7 (11)	0.06
26. I was able to reach students easily	3.0 (11)	4.4 (11)	0.02*
27. Leading online/on-site plenary discussions went well for me	3.7 (11)	4.4 (11)	0.09
28. I did NOT find TBL online/on-site to be stressful	4.1 (11)	4.4 (11)	0.34
29. I found TBL online/on-site exhausting	3.2 (11)	2.9 (11)	0.23
30. In general, I grade my role as a TBL teacher online/on-site to be.	3.8 (10)	4.2 (10)	0.20

Averages of 1–5 Likert scales (1 = completely disagree to 5 = completely agree) are shown. *p* values were from paired *t*-tests, non-normal distribution, Wilcoxon signed rank test. *p < 0.05.

Qualtrics was used as the platform for the online questionnaires, which were made available through a link in the digital learning environment Canvas for students, and through an e-mail containing the link to the eligible teachers. Participants were allowed to cease participation at any time while filling out the questionnaire. All participants contributed anonymously.

#### 2.3.2 Interviews

Students and teachers were interviewed to add depth to the survey findings. In these interviews, we explored in greater detail how students and teachers perceived the transition, the challenges encountered in online and on-site TBL, and the extent to which specific elements of online TBL might contribute added value to on-site TBL. Both interviews were semi- structured. Teachers were interviewed as group, students on two occasions: one interview with an individual student and one interview with two students. Teacher group interviews were held on-site while the interviews with students were held online in a secure environment in Zoom. Each interview lasted about 60 min. The interviews were conducted by the second and the last author (KG and GS). Semi-structured interview guides, specifically designed for this study, were used for the interviews with students and teachers. The individual interviews and the group interviews were structured around themes similar to the questionnaires. The group interviews with the teachers were audio recorded while the interviews with students were captured with Zoom’s audio-visual recording function.

### 2.4 Research setting

The undergraduate program at ACTA has a curricular structure that includes traditional lectures, self-study and small group instructions.

At ACTA, Team-Based Learning (TBL) was implemented in 2020 as a new structural component for all courses of year one of the undergraduate program. For both the on-line and onsite versions of TBL, ACTA followed the general principles of TBL as laid down in (16). Each course offers one TBL module. Each TBL module consists of three phases: the preparation phase (self-study), the readiness assurance phase (iRAT, tRAT and mini lecture), and the application phase. The preparation phase is an individual study phase. In this phase students obtain new information by completing scheduled activities such as readings or e-learning assignments (17). The information they obtain during this phase is necessary to be able to engage in the learning activities in the subsequent phases. In the readiness assurance phase, students take two tests (iRAT and tRAT) to measure their overall understanding of the concepts and knowledge that they need to apply in the application phase. Within this structure, iRAT and tRAT are assessed in a summative way. At the end of this readiness assurance phase, students receive instructor feedback on concepts they did not understand. Finally, in the application phase, students share and apply the learned information of the previous phases to solve problems (‘application exercises’) (18). The application exercises follow the four S″ principle (4):

- *Significant problem*: Students solve realistic problems.
- *Same problem*: All teams work on the same problem.
- *Specific choice*: Each team must make specific choices after intra-team discussion,
- *Simultaneous report*: Teams present their answer to a specific question simultaneously in the intergroup discussion.

A typical application exercise lasted 1.5 h, during which there were three application exercises; each application exercise could be answered by choosing one of 4 alternatives (A-D).

TBL started entirely online in September 2020. To conduct and monitor the iRAT tests Testvision and Proctorio were used; for the tRAT, Zoom’s breakout rooms were used. TestVision is a tool for (summative) digital assessment, which can be used both online and on campus. Proctorio is a tool that monitors students while they take an online home exam by sharing their screen and using their webcam. Zoom is a digital platform to organize video conferences, webinars, and chat sessions. The application phase took place online using Zoom and breakout rooms, lasting approximately 1.5 h. For this, the cohort of 24 teams was split into 4 groups of 6 teams, each team consisting of 6 students. The teams were established at the start of the academic year randomly and remained together throughout the academic year.

At ACTA, peer evaluation differs from the approach found traditionally in TBL. Instead of using both a qualitative and quantitative component serving formative and summative purposes (4) ACTA has chosen to train students to provide oral, narrative feedback on each other’s contribution to the TBL process. This feedback is intended to encourage improvement and does not serve a summative function.

For the t-RAT and application sessions, students were placed by breakout room into their teams. For working on the application exercises, students were placed in breakout rooms, so that each team had its own space to work independently and undisturbed. The teachers randomly joined the teams to facilitate the team discussions. After discussing within their teams, students returned to the plenary session and simultaneously answered the question prepared in the breakout room using Zoom’s chat function. Teachers facilitated the plenary discussion between teams, reflected on the discussion between teams and provided feedback to the teams on the TBL-process and on the outcome of the TBL-process.

The academic year that followed, 2021–2022, TBL was still entirely online, except for the iRAT, which was performed on-site at a test center outside ACTA. One adaptation this year was that Proctorio was no longer utilized after filling out the iRAT, students had to return home to complete the tRAT together via Zoom and breakout rooms. This version of online TBL is referred to in the student questionnaire.

From the academic year 2022–2023 onwards, TBL took place on-site. This time, students took part in their existing team of six people on-site. The iRAT was performed on a test computer with vertical screens separating students to prevent cheating. After the iRAT, the separating screens were taken away so students could discuss and fill out the tRAT together, using one of the computers. During the on-site application phase, 12 teams participated in the first round, while the remaining cohort was scheduled to participate later the same day.

In both scenarios—responding via Zoom’s chat or displaying colored letters on a stand—the TBL principle of simultaneous answers was maintained.

**Figure 1:**
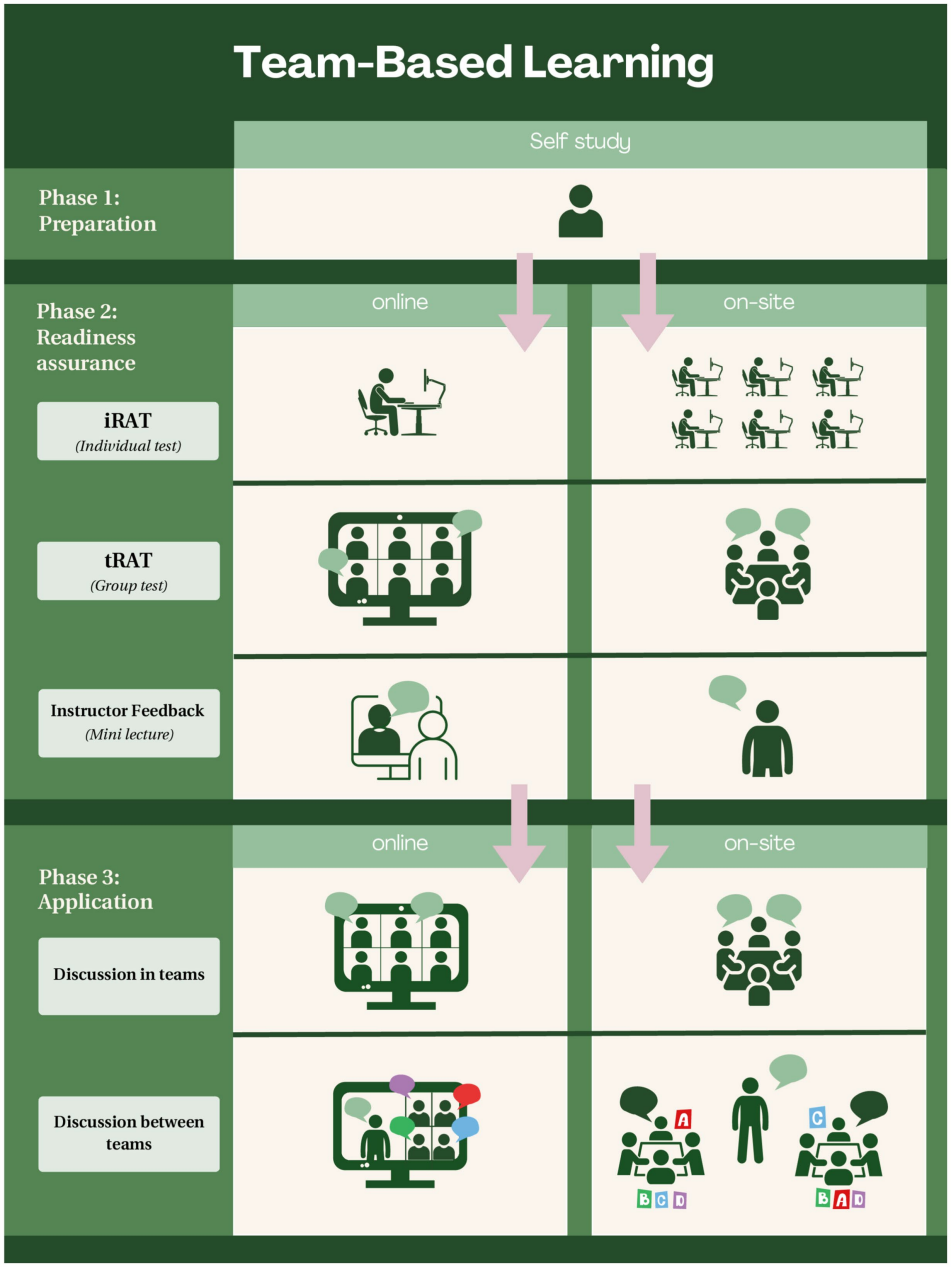
Schematic representation of online and on-site TBL. The left side of the diagram displays the process of online TBL and the right side displays the process of TBL on-site. See text for details.

In Figure 1, the processes of online and on-site TBL are displayed as a diagram.

### 2.5 Ethics

Participation in the study by teachers and students was voluntary. Students and teachers were informed about the purpose and the use of the study. Students and teachers received an informed consent form with the invitation to participate. Recordings of the interviews were only made if members had given their permission for these recordings. Furthermore, students and teachers were informed that data analysis would be confidential, the data presentation anonymous, and that all privacy protection rules would be followed. In addition, students were assured that neither their participation in the study nor the research results would affect their course grades. Additionally, students and teachers autonomously decided whether to participate in the survey and the interviews. Approval for conducting the study was obtained from the local Ethical Review Committee of ACTA under number 2023-12397.

### 2.6 Analysis

#### 2.6.1 Questionnaire data

A paired *t*-test was used to analyze the outcomes of the 1–5 Likert scale questions. Since the answers were not normally distributed, a Wilcoxon signed rank test was used as a ranked post-test.

#### 2.6.2 Interview data

All recorded interviews were transcribed verbatim and analyzed using a thematic analysis approach in six phases (19). To focus primarily on the information that students and teachers, an inductive approach was adopted, meaning that the codes and the themes were derived from the data rather than linking them to existing theoretical frameworks.

## 3 Results

### 3.1 Students and TBL online versus on-site

#### 3.1.1 Participants

Students were all in Bachelor-2. This unique cohort started entirely online in their first year followed by onsite in the second year, which means that they all had experience with approximately 10 online and 10 on-site TBL-modules. The questionnaire was filled out by 54 students (response rate = 40%). Of the respondents, 8 identified as male, 45 as female and 1 as non-binary which nearly resembles the overall gender distribution in the Bachelor’s degree program. The average age of the respondents was 23.0 years, similar to the cohort.

Three students participated in individual interviews and two group interviews were carried out with teachers (*N* = 5).

#### 3.1.2 Results of the student questionnaire

The 37 questions were divided into the three phases of TBL (preparatory self-study, readiness assurance test (RAT), application), the role of teachers, and student engagement. All questions and responses are listed in Table 1. One question on student participation during TBL scored significantly higher (*p* = 0.02) for the on-site than the online version.

#### 3.1.3 Student interviews

Following the questionnaire, we conducted interviews with students to gain deeper insight into their experiences with the transition from online TBL to on-site TBL.

During the analysis of the interview data, subthemes were identified and classified under four major themes: flexibility, learning environment, engagement and integration. These themes are spread over the results section. Furthermore, the themes are presented in Table 3 in combination with a summary of the most important findings of the results of the analysis of the student interview data.

**Table 3:** Central themes and a summary of the student and teacher perspectives on online vs. on-site TBL.

Central themes	Student’s perspective	
	Online TBL	On-site TBL
Flexibility	No travel time and TBL materials are easily accessible	Teachers are more accessible for feedback
Learning environment	‘Quiet’ learning environment with focus on TBL application exercises	More disruptions on the one hand, easier communication and better discussion
Engagement	Propper student led discussions	More engagement in the learning process, especially in the TBL application sessions
Integration	Sense of ‘TBL group integration’	Helpful for ‘broad’ social and academic integration

Central themes	Teacher’s perspective	
	Online TBL	On-site TBL
Flexibility	No travel time; information sharing is easy	Deeper insight into the TBL learning process on what basis feedback can be given more appropriately
Learning environment	Structured, quiet monitoring in breakout rooms	Deeper insight into student and group processes and what students need by walking around in the TBL-room; especially in the TBL application sessions
Engagement	Teams seem to be engaged in breakout rooms	Better and more dynamic interaction with and between students
Integration	Sense of ‘TBL group’ integration	Promotion of social and academic integration

##### 3.1.3.1 Transition from online to on-site TBL

Students indicated that the transition from online TBL to on-site TBL proceeded ‘smoothly’, with scheduling misalignments, caused by rooms and lecturer availability, being the only hurdle. As one student put it (S1),

‘It’s just that it is always split into two groups and if you are in the afternoon group, you are just hanging around at the university until you can start. You have less of that when TBL is offered online. Then you take a break at home before you get back to work.’

Moreover, the students experienced the TBL days on-site as more demanding: assessments, mini lectures, a gap in the schedule of sometimes 2 hours, and then the application sessions.

##### 3.1.3.2 Challenges in online and on-site TBL

###### 3.1.3.2.1 Preparatory self-study

Students indicated that they did not prepare differently for online and on-site TBL. However, they did indicate that it is easier to ask the instructor which study materials are particularly relevant in preparation for the TBL tests and the TBL application sessions during on-site TBL. As one of the students put it (S1):

‘It is [in on-site TBL] a bit easier to ask teachers which material is important to study in advance. You do not do that online as easily.’

###### 3.1.3.2.2 TBL-assessment

Students did not experience any advantages from the combination of iRAT on-site and tRAT online. They expressed a preference for both the iRAT and tRAT on-site as the collaboration during the online tRAT using Zoom’s breakout-room option was less smooth. Or as one student (S2) put it:

‘In an on-site tRAT, it’s easier to ask fellow students what they think. And if someone has their camera off or is very quiet, you think we should move on. It’s easier to involve someone on-site, which does not always work either, but it is easier. Or like, does everyone agree? Okay, let us move on. Online, that is more difficult; everyone has to unmute, and it takes a bit longer.’ The online tRAT also came with more stress for students. As one of the students (S1) described it, ‘For me, another advantage of being on-site and not online is that I do not have computer stress with my wifi and Testvision. So, I’m happy that is not an issue anymore.’

###### 3.1.3.2.3 TBL mini-lecture

Students indicated that there was no difference in effectiveness between offering the TBL mini-lecture online or on-site. However, when students already had to be on-site for other TBL activities, offering the TBL mini-lecture online was not convenient in terms of scheduling, students reported. On the other hand, sharing information online was easier than on-site, according to the students.

###### 3.1.3.2.4 TBL application sessions

In the online TBL application sessions [the break-out rooms], discussions with team members sometimes went less smoothly. Unmuting always caused disruptions in communication and was also experienced as an extra step to contribute to the discussion (S1).

‘As I said before, unmuting is an extra step you have to take to say something. Sometimes you think, oh never mind. Whereas if you are actually sitting across someone, you think, let me share my opinion.’

Or as another student put it,

‘I do think the barrier is indeed larger online. When people are talking, you think, I’ll just stay out of it. And I think when you are together, you can interfere more quickly to say something.’

Furthermore, students indicated that in the break-out rooms, they were more likely to be occupied with other things than the TBL application exercises. Or as a student (S2) indicated:

‘Because in the breakout rooms it was often like, hey guys what are you going to do this afternoon? And then suddenly you saw a teacher step into the breakout room, and it was like uujaa so uhhh. That happens much less on-site.’

Students perceived that team members were less prepared for online TBL than for on-site TBL. Or as one student (S1) put it:

‘But I do think that people are inclined to prepare better on location than online. Online they can always say that the connection is poor, that other students are hard to understand, to turn off the camera, etc. On location, that is difficult; you still want to say something, convey something, I think.’

Furthermore, students considered teacher contact to be easier on-site, particularly when they had questions about the material. As one student (S2) put it:

‘You can easily approach the teacher afterwards on location to ask why it wasn’t answer B, and then you can get a bit more clarity about it. You do not do that online.’

The discussions at the end of the online TBL application sessions with all participating teams were conducted in a more structured manner than on-site.

Teachers still had to adapt to facilitating such discussions, as one of the students (S1) put it:

‘On-site, the discussion between teams often becomes a bit messy, leading the teacher to suddenly say answer A is the best, but answer C could also be valid. Then someone else asks a different question in between about how exactly things are, and it gets messy. In the online version, it was much more structured.’

###### 3.1.3.2.5 Learning outcomes

Students indicated that the learning outcomes of TBL were not determined by its format, but rather by team functioning. They emphasized that effective teamwork primarily depends on the content-related preparation and their motivation to fully engage or as one student (S1) put it:

‘It depends on whether your team is motivated to perform well as a team and the preparation for the iRAT by the team members. I can understand when some team members are not fully familiar with the material yet. And if someone is not well prepared, you can learn less from that person because you are not on the same level in terms of what is in your head. I notice this sometimes.’

This observation was confirmed by other students. When asked whether students discuss this, they noted that it is more difficult online due to limited interaction. Or as one of the other students (S2) put it,

Face to face it is easier to talk about something like this,’ or as yet another student (S3) put it:

‘Yes, personally I find it harder to talk to someone when you are behind the camera.’

##### 3.1.3.3 Added value of online TBL elements for on-site TBL

The survey revealed that some students were more bothered by the noise and stimuli during the on-site TBL sessions which was confirmed during the interviews by one of the students (S1):

‘If it were a little less noisy, if the room could be somewhat isolated so that you can consult without being distracted by all the chaos around you, that would be nice.’

At the same time, students indicated that this might be difficult to achieve in practice because it would require working with a smaller number of TBL teams in a smaller TBL room.

### 3.2 TBL teachers and TBL online versus on-site

The teacher questionnaire was filled out by 11 teachers (response rate = 57.8%). Of the respondents, 6 identified as male, 5 as female. Average teaching experience was 19.3 years (s.d. = 12.8, range 4–40).

#### 3.2.1 Results of the teacher questionnaire

The results from the 30 questions for teachers, divided by TBL phase (preparatory, readiness, application), and their role as teacher, are listed in Table 2. Interestingly, despite the lower number of participants, teachers were more articulate on a preference for on-site than students (teachers: 7 out of 30 paired questions favored on-site; versus 1 out of 37 questions for students) and clearly favored the on-site-variant. A Wilcoxon signed rank test indicated significantly higher scores for 7 out of 30 paired questions related to the online version compared to the on-site version of TBL. Five of these questions concerned the RAT-phase.

#### 3.2.2 Interviews with teachers

As with the students, the teachers’ questionnaire was followed by interviews with TBL teachers to discuss their experiences with transitioning from online TBL to on-site TBL, both in terms of challenges and positive aspects of online TBL to add value to on-site TBL.

During the analysis of the interview data, subthemes were identified and classified under four major themes: flexibility, learning environment, engagement and integration. These themes are spread over the results section. Furthermore, the themes are presented in Table 3 in combination with a summary of the most important findings of the results of the analysis of the teacher interview data.

##### 3.2.2.1 Transition from online to on-site TBL

Almost all teachers indicated that they experienced the transition as fairly smooth. Both students and teachers had only prior experience with online TBL, which may have made the transition to on-site TBL easier, since the basics of the teaching method of TBL were well introduced at ACTA. Furthermore, the logistical support on-site was well organized, as was the ICT support, which laid a solid foundation for the implementation of TBL. However, teachers indicated that the support for the on-site version of TBL will remain necessary in the future. Or as one teacher (T1) put it:

‘[the on-site RAT phase] requires quite a lot of support. I see that it is there now, but it cannot fall away. But also, the fact that all those computers are ready, the flags with ABCD, all those logistical aspects around it, I assume that there is quite a bit of work involved, you can’t ask the individual coordinators to do that.’

The majority of the teachers also indicated that the energy was almost immediately ‘better’ when TBL was offered on-site, which smoothened the transition or as one of the teachers (T4) put it:

‘I find the energy much better on location. You can really see those groups working. You can also see much better that they are engaged with the assignment, that a discussion evolves. Of course, I used to visit them in breakout rooms, but I do not know what’s on their screen, and I feel that more discussion can occur. Using a laptop, they are sort of lounging on their bed.’

Only a few teachers indicated that they needed to get used to the TBL room and the structuring of the TBL activities and missed the calmness of and the switching between breakout rooms. Or as this teacher (T2) stated:

‘The online TBL was very structured. You could easily switch to a breakout room and also listen along quietly. Often without them realizing that you were there, so you could monitor what the dynamics were.’

##### 3.2.2.2 Challenges in online and on-site TBL

###### 3.2.2.2.1 Preparatory self-study

Teachers indicated that they intuitively felt that students did not prepare differently for online TBL compared to on-site TBL, although they had no concrete insight in what the students actually did in either situation. Or as one teacher (T2) said:

‘I have no overview of how they prepare, but I have not noticed any difference either. It also seems that students do not prepare less effectively for TBL online than for TBL on-site. I have not noticed any differences in their quality-levels either.’

This observation was confirmed by the other TBL instructors. Additionally, teachers reported receiving questions more regularly about the preparatory self-study during on-site TBL, particularly regarding the study material that students needed to master for the tests and the application sessions, which led to improved communication about this. Or as one other teacher (T5) said:

‘And at some point, you start getting questions from students like, do I need to know this or not. Following this question, I made the study material much clearer on Canvas after that.’

###### 3.2.2.2.2 iRAT and tRAT

Teachers indicated that the combination of an on-site iRAT with an online tRAT was unfortunate. For students, it was unfortunate because they had to go home to do the online tRAT after doing the on-site iRAT. For teachers, there were concerns about cheating when taking the online tests (including proctoring). As T4 said:

‘Proctoring? I think we were really fooled during that time. Yes, I suspect so.’

Another TBL teacher (T3) added:

‘The internet is full of tips on how to bypass proctoring.’

Teachers also indicated that students may not always have the means to take a test at home or follow online application sessions, causing stress or discomfort when working with the camera on. As one teacher (T4) mentioned:

‘I can imagine that many students, given that they did not really have a good workspace at home, were in a family with so many children that they did not even have a good place to sit anywhere. And when you then are tested, that can be very unpleasant. Of course, we should be considerate about this. I always think from my own history, and my own room with my own computer. But that is not the reality for some of our students. I have seen this in various online activities, that some people do not always have the privacy they need for testing.’

The on-site assessment went satisfactorily after some initial hiccups, especially thanks to the IT services and logistical support from ACTA. Teachers expressed that they were satisfied with the on-site administration of the tests. Although some students complained about noise from other teams during the on-site tRATs, this was not observed by the teachers. However, they noticed it in the application sessions or as one teacher (T2) said:

‘Then you could see that there were groups that finished faster, and they would interfere with other groups. And I can imagine that students are disturbed by that.’

###### 3.2.2.2.3 TBL mini-lecture

Teachers mentioned both pros and cons of the TBL mini-lectures online versus on-site and did not favor either format. Some teachers indicated that it was easier to engage students in the follow-up discussion about remaining questions during on-site lectures. However, it was also more difficult to share information in the on-site version. As one teacher (T1) mentioned,

‘For me it was easier online, because we received the results of the test at home, and I could just pull up a few slides while sitting at my computer. And being on-site was a bit more difficult, as you were in the same room as the students. That’s how I experienced it; of course, I could have sat somewhere else. My experience was that it was more comfortable to work from home than being there on-site. And that room was not suitable for presentations; there was a small screen, but the room was too large to accommodate it.’

###### 3.2.2.2.4 TBL application sessions

Teachers indicated that students collaborated well during the online TBL application exercises but they still preferred the on-site TBL application sessions. For example, one teacher (T4) mentioned that it was ‘easier to engage students by approaching them when they were working in teams while walking through the room.’

The contact and interaction with students were different during on-site application sessions compared to online application sessions. As one teacher (T3) noted:

‘You missed the non-verbal communication in the online setting.’ Another instructor stated that during the on-site sessions, ‘You could sometimes see from a student’s face who understood it and who did not, and you could then act upon that, which you missed online.’

Another teacher (T5) noted in this context:

‘I found it more difficult to know whether everyone was actively participating in the online application session, and by the end of such a session, I felt a bit empty myself; you put a lot of energy into it and you did not know if it landed, and I did not get any energy from it. For me, it was more about giving, let us put it this way, it was structured, but I did not know, if they were all on mute, what they were actually doing. And when I was explaining: were they listening to me, or chatting with each other? Sometimes there were also comments coming in the chat and then it was oops sorry that was meant only for me.’

The teachers unanimously emphasized that on-site TBL application sessions provided valuable opportunities to gain direct insight into team activities and to observe and stimulate student participation when needed. It was also easier to encourage students and student teams to stay engaged and to provide support when they were stuck on the assignment or as one teacher (T4) mentioned:

‘I found it easier to see the group process and to hop between groups and listen to the content and then do something with it, than when you walk by in such a larger context; whenever you are there, they tend to behave differently.’

By circulating in the TBL room with multiple teachers, the teachers perceived that they could more effectively involve students and student teams, sustain engagement, and maintain focus on the task. Or as T2 indicated:

‘The student’s attention, I have to say this correctly, is inversely proportional to the distance from the teacher.’ You could also just give whole teams a turn, then they [students] know ‘I cannot opt out because at any moment a table could be getting a turn.’ In this context, T4 added ‘I feel that by walking around I had a better overview of what students were working on, and if students were on the wrong track, I would stay and then, for example, try to give more voice to the students who understood it. That really went less well online.’

Teacher 3 (T3) followed up on this:

‘During on-site TBL you got a better view of how the thought processes were going and whether you needed to adjust. If you had a group that had no idea about what was going on, which there of course always was, they were identified more quickly so that you could intervene better as a teacher.’

Regarding teacher support during the online TBL application sessions, teachers noted the added value of co-facilitation. As one teacher (T4) put it:

‘It was handy to work in pairs, but it was also more practical because, as we said, one person was busy and the other could check attendance and cameras and go around to some of the groups in the breakout rooms while the other teacher took care of the other groups. So, it was more practical in that way.’

Furthermore, teachers indicated that student participation during the interaction between teams at the end of the TBL application session was more intense and dynamic, and more students participated during the onsite application sessions compared to the online application sessions, as one of the teachers (T4) put it:

‘During the larger follow-up sessions at the end of the TBL application sessions, you could see that more students withdrew when the session had an online character. Then you saw that only a limited number of students interacted with each other.’

Likewise, it was mentioned by another teacher (T2):

‘Working in the breakout rooms went quite well, but when they returned for the debriefing, it was definitely nicer to do it on location rather than online where you had a large panel with all those screens and where you noticed that students were a bit slower to provide input.’

Yet another teacher (T5) stated in this context:

‘But it was more about discussing that application assignment of what you had as a group and why you have chosen A and you did not. (…) That action, that dynamic that arose, I believe is valuable. Looking at different viewpoints together, discussing them, and then arriving at new insights. I think that works better in a physical setting.’

###### 3.2.2.2.5 Learning outcomes

Although teachers mentioned several advantages of on-site TBL over online TBL, they did not know whether this also led to better learning outcomes, or as T3 indicated:

‘In terms of learning outcomes, on-site TBL does not stand out.’

All teachers agreed with this. At the same time, TBL teachers stated that the specific learning outcomes of TBL are not measured, which makes it difficult to say anything about it.

##### 3.2.2.3 Added value of online TBL elements for on-site TBL

Although teachers did not indicate to miss many elements of online TBL during on-site TBL, they did think that it was easier to share information and provide feedback during the online TBL mini-lecture and the plenary inter-team discussions. As one teacher (T5) noted:

‘Online, it was easier to share a screen demonstrating an explanation using an image. So when we talked about something and you wanted to clarify it with an illustration, that was easier online.’

##### 3.2.2.4 Online or on-site preference per TBL category

Finally, we analyzed the outcomes of all questions per category, both for the students (Table 1) and teachers (Table 2). The Likert-scale outcomes of all questions per category were analyzed. They were generally higher for on-site, therefore with the applied Wilcoxon signed rank test, 4 categories (two for students, two for teachers) scored higher. Results indicate that both students and teachers were in favor of the on-site version, especially during the RAT phase. Additionally, teachers appreciated the on-site version of the application phase of TBL. Students felt more engaged with the on-site version of TBL (Table 4).

**Table 4:** Summary of student and teacher questionnaires with responses to the five categories.

Phases of TBL	Student	Teacher
Preparatory self-study (4 questions)	0.13	0.25
RAT phase (11 questions students and 9 questions teachers)	0.01*	0.01*
Application phase (11 questions)	0.14	0.01*
TBL-teacher (5 questions)	0.06	0.19
Student engagement (6 questions)	0.03*	-

*For all *p* values < 0.05: where significant there was preference for TBL on-site. Testing: paired *t*-test, non-normal distribution, Wilcoxon signed rank test.

### 3.3 Online or on-site preference

The final question of both questionnaires asked students and teachers explicitly whether they favored the online or on-site version. There was a clear preference for on-site (*p* < 0.0001, The Chi-square test) indicated a strong preference for onsite (*p* < 0.0001) especially among teachers, all of whom favored on-site over online (Table 5).

**Table 5:** Preference for online or on-site.

Preference	Student	Teacher
Online	8 (26.6%)	0 (0%)
On-site	20 (66.7%)	8 (72.7%)
No preference	2 (6.7%)	3 (27.3%)

In the free-text field of the questionnaire, where students were able to explain their choice for either online or on-site, one of the pro-online students remarked (S1):

‘Honestly, I find on-site more enjoyable and I stay more focused, but I still prefer online because I prefer to study at my own pace and from home rather than attending lectures.’

The pro-on-site students gave as feedback:

- ‘On-site, TBL is much more lively, active, and educational. More people participate, you stay active, take everything in, and it is just nicer when it is ‘in person,’ so to speak.’ (S3)

A selection of the teachers’ feedback:

- ‘TBL on-site is much clearer, better for discussion.’ (T1)
- ‘Both on-site and online versions have strong points.’ (T2)
- ‘On-site interaction is better.’ (T3)
- ‘Being able to look each other in the eye, sense the atmosphere, and see and discuss any problems early on. Much better on-site than online.’ (T4)
- ‘I find TBL on-site not comparable to TBL online, in the sense that I disapprove of TBL online. It is a farce to think that students actively participate.’ (T5)
- ‘Better contact with the students, good logistics. Also, nice that there is didactic support from the TBL team.’ (T6)

### 3.4 Summary of the results

Table 3 presents the four major interview themes in combination with a summary of the various aspects of both student and teacher perspectives on online vs. on-site TBL.

## 4 Discussion

### 4.1 Main outcomes

Globally, education has shifted back from online to on-site learning after Covid-19 thereby addressing students’ social isolation (20, 21) and lack of belonging within the academic environment. However, students who began online had to adjust to face-to-face classes. This study examined the transition from online to on-site TBL by exploring (1) experiences of the transition; (2) challenges faced by students and teachers; (3) good online practices that inspire on-site TBL.

The overarching aim of this research was to explore the transition process from online to on-site TBL. Although the transition was described as smooth, participants predominantly discussed the benefits associated with on-site in comparison to its online counterpart.

A closer look revealed that teachers especially preferred on-site, echoing previous research by Yu (11) that found that students were more comfortable online than teachers. However, both students and teachers agreed that especially the RAT-phase was better on-site, with teachers favoring the on-site application-sessions and students feeling more connected to each other in the on-site application sessions. Online TBL provided students a sense of belonging to a group, which is essential for initial academic engagement when everything is online. Furthermore, the online break-out room was appreciated which confirms previous research on the online version of TBL (3). Thus, online education could be an alternative for some students.

The results of this study are of special interest for active learning methods such as TBL, which encourage collaborative group work, preparation of academic reasoning, and decision making (22). Unsurprisingly, the study shows that on-site collaboration work formats, specifically the (t)RAT (both teachers and students) and the application session, were valued more highly than the online version. Compared to our earlier evaluation of the introduction of online TBL (3), students rated TBL’s benefits for collaborative work even higher in the on-site format.

Thus far, most studies have addressed the transition from on-site to online (e.g., 8,9), finding, e.g., that students adapt better to online formats than teachers, who feel less confident delivering TBL remotely (11). Both groups reported technical hurdles, changes in delivery, reduced student engagement, and difficulty focusing in class as significant challenges in the online transition. In another study, where online TBL was optional during Covid-a9, students welcomed the choice, with no major differences between the online and onsite format (12).

Our study found that teachers were more vocal about their preference for on-site than students. No teacher selected online as their preferred format, which was possibly due to their previous experience with various face-to-face teaching methods before Covid-19. In contrast, many students (27%) favored online TBL, reflecting their adaptability from starting university completely online. Our findings align with those of Nikoupoulou (23), where about a third of all students preferred hybrid or online learning, and with a South Korean study (24), in which 84% of the medical students preferred online teaching, compared to just 14% of the teachers.

### 4.2 Limitations and strengths

Kirckpatrick’s model for evaluating educational innovations (25) includes four levels: reaction, learning, behavior and results. This study assesses level 1 (reaction), focusing on engagement, relevance and satisfaction, using a mixed methods approach. This approach, evaluating both students´ and teachers` perceptions through a quantitative questionnaire as well as qualitative interviews, is the major strength of the present study (14, 26). The questionnaire framework which had previously been used for addressing the educational aspects of TBL (15), was now used to compare online and on-site learning at ACTA. This adds validity. Finally, the large total number of questions (30–37) and the number of questions per theme (7, 8) allowed for detailed responses, while identical questions for both formats enabled a statistically stronger paired analysis.

This study’s limitations include the low number of students that were interviewed and the student questionnaire response rate that was not high. However, based on the age and the gender distribution of the response group, this group seemed quite representative of the total population and it can be cautiously concluded that the survey data are reliable. Finally, we did not compare test performances between the online and on-site format due to large differences between the bachelor-1 and bachelor-2 courses. However, other research in which face to face teaching was followed by a comparable online module (27), indicates that test results do not depend on the ‘delivery’ method, though students prefer face-to-face teaching (28).

The decision to redesign TBL as an on-site version was partially ignited by the observation that students who had started their studies entirely online (29, 30), were not used to face-to-face teaching (30). Sense of belonging may be strengthened when feeling socially attached to the learning environment (16). The present study clearly shows that the transition has worked out well, both for students and teachers.

Based on a questionnaire and interviews with students and teachers transitioning from online TBL back to on-site TBL, this study found that both teachers and students preferred on-site TBL for all phases (preparatory self-study phase, readiness assurance test (RAT) phase, and the application phase) of this learning approach. They considered interaction among students, as well as between students and teachers, to be more meaningful during on-site TBL. Furthermore, students found the on-site RAT phase quieter, while teachers appreciated that fraud was less likely to occur during on-site RAT. The only element of online TBL that was appreciated by both students and teachers, was the mini-lecture, because it is easier to share information during this online lecture than during an on-site lecture. Based on our research findings, as well as similar findings of several other studies [e.g., (31–33)] we suggest that a blended approach to TBL could be considered, warranting further research on both the advantages and disadvantages of such an approach. The unknown normal has been explored and has a lot to offer for the future.

## Data Availability

The original contributions presented in the study are included in the article/supplementary material, further inquiries can be directed to the corresponding author.
